# miR-146a-5p inhibits TNF-α-induced adipogenesis via targeting insulin receptor in primary porcine adipocytes[S]

**DOI:** 10.1194/jlr.M062497

**Published:** 2016-08

**Authors:** Di Wu, Qian-Yun Xi, Xiao Cheng, Tao Dong, Xiao-Tong Zhu, Gang Shu, Li-Na Wang, Qing-Yan Jiang, Yong-Liang Zhang

**Affiliations:** National Engineering Research Center for Swine Breeding Industry, Guangdong Provincial Key Lab of Agro-Animal Genomics and Molecular Breeding, College of Animal Science, South China Agriculture University, Guangzhou, China, 510642

**Keywords:** microarray, pig model, Gene Ontology, insulin receptor substrate-1, microRNA

## Abstract

TNF-α is a multifunctional cytokine participating in immune disorders, inflammation, and tumor development with regulatory effects on energy metabolism. Our work focused on the function of TNF-α in adipogenesis of primary porcine adipocytes. TNF-α could suppress the insulin receptor (IR) at the mRNA and protein levels. Microarray analysis of TNF-α-treated porcine adipocytes was used to screen out 29 differentially expressed microRNAs (miRNAs), 13 of which were remarkably upregulated and 16 were intensely downregulated. These 29 differentially expressed miRNAs were predicted to mainly participate in the insulin signaling pathway, adipocytokine signaling pathway, and type 2 diabetes mellitus pathway by Gene Ontology and Kyoto Encyclopedia of Genes and Genomes analyses. miR-146a-5p, reportedly involved in immunity and cancer relevant processes, was one of the most highly differentially expressed miRNAs after TNF-α treatment. Red Oil O staining and TG assay revealed that miR-146a-5p suppressed adipogenesis. A dual-luciferase reporter and siRNA assay verified that miR-146a-5p targeted IR and could inhibit its protein expression. miR-146a-5p was also validated to be involved in the insulin signaling pathway by reducing tyrosine phosphorylation of insulin receptor substrate-1. Our study provides the first evidence of miR-146a-5p targeting IR, which facilitates future studies related to obesity and diabetes using pig models.

Adipocyte development is an essential process of energy metabolism. With advanced research, adipose tissue is no longer considered merely a storage depot but is also known to function as an important endocrine organ (1). Dysfunction of adipose tissue will lead to a series of metabolic syndromes involving insulin resistance, type 2 diabetes, cardiovascular disease, and fatty liver disease (2). Pigs have served as a major mammalian model for human studies because the two species are similar in size, physiology, organ development, and disease progression (3). Moreover, porcine adipose tissue contributes to the quality of pork in animal husbandry (4). Thus, a better understanding of adipose tissue regulation benefits both animal husbandry and human health.

MicroRNAs (miRNAs), a class of highly conserved small noncoding RNA, participate in numerous biological processes by regulating mRNA translation at the posttranscriptional level (5). miRNAs are reported to play important roles in adipose tissues, including adipogenesis (6–14), lipid metabolism (15–20), insulin resistance and diabetes (13, 21–24), inflammation (6, 25, 26), and paracrine and endocrine communication (27–29).

TNF-α has been long been known to be at the center of immune disorders, inflammation, and tumor development (30), and subsequent research has revealed that it also functions as a pivotal regulator in energy metabolism. As a multifunctional cytokine that is expressed in and secreted by adipose tissue (31), TNF-α is involved in almost every aspect of adipose biology (32). In terms of adipocyte differentiation, TNF-α inhibits adipogenesis by preventing the early induction of the adipogenic transcription factors PPARγ and CCAAT/enhancer binding protein-α (C/EBPα) (33). Important genes responsible for the mature adipocyte phenotype, such as aP2, glucose transporter type 4 (Glut4) and adipose triglyceride lipase (ATGL), are also downregulated following TNF-α treatment (34, 35). Even though the mechanism of TNF-α-induced suppression of adipogenesis has been well studied, studies identifying miRNAs involved in this process are lacking.

In the present study, we treated porcine adipocytes with TNF-α and screened differentially expressed miRNAs by miRNA microarray. Interestingly, miR-146a-5p was detected to be highly upregulated after TNF-α treatment. miR-146a-5p has been reported to participate in the immunoregulatory activity of bone marrow stem cells (36), proliferation and migration of mesenchymal stem cells (MSCs) (35), gastric cancer (37), and lipopolysaccharide-mediated tolerance (38). In this study, we investigated the function of miR-146a-5p in the process of adipogenesis. Insulin is a key hormone regulating adipogenesis that acts by binding to the insulin receptor (IR), thereby activating its tyrosine kinase activity and resulting in phosphorylation of insulin receptor substrate (IRS)-1 and IRS-2 (39). miRNAs targeting the IR have not been reported previously, and our study is the first to verify that miR-146a-5p targets IR.

## MATERIALS AND METHODS

### Ethics statement

All the animal experiments were conducted in accordance with the guidelines of Guangdong Province on the Review of Welfare and Ethics of Laboratory Animals approved by the Guangdong Province Administration Office of Laboratory Animals. All animal procedures were conducted under the protocol (SCAU-AEC-2010-0416) approved by the Animal Ethics Committee of South China Agricultural University.

### Sample collection and culture of primary porcine preadipocytes

Subcutaneous fat tissue from a 7-day-old piglet was isolated aseptically and transferred to DMEM-F12 nutrient mixture (GIBCO, Grand Island, NY). After removing the visible connective tissues, the adipose tissue was cut into small pieces of about 1 mm^3^, and the subcutaneous preadipocytes were obtained as described in previous reports (40). Minced tissue was transferred into a Carlsberg’s flask, digested in 0.2% type-II collagenase (1 mg/ml, GIBCO) for 2 h at 37°C, and then filtered through a 150 μm mesh. Cells in the filtrate were centrifuged at 500 *g* for 10 min, and erythrocytes were lysed using erythrocyte lysis buffer (0.154 M NH_4_Cl, 10 mM KHCO_3_, and 0.1 mM EDTA). After filtering through a 40 μm mesh, cells were rinsed with F12 and centrifuged at 1,500 *g* for 5 min. The preadipocytes were collected and plated in growth medium.

### TNF-α treatment and induction of primary porcine preadipocytes

Preadipocytes were cultured in 6-well plates and induced to mature adipocytes with induction medium (10% FBS, F12, 50 μM oleic acid, 0.5 mM octoic acid, 50 nM insulin, 50 nM dexamethasone; reagents were purchased from Sigma Co). For phosphorylated IRS-1 (phospho-IRS-1) Western blot detection, preadipocytes were kept in serum-free medium for 3 h after 8 days’ induction and stimulated with 100 nM insulin for 30 min at 37°C (41, 42). TNF-α (100 ng/ml, PeproTech Inc.) was added to the induction medium starting from induction day 2 until cell collection; the same amount of DMEM-F12 was added to control group. The dose for TNF-α-induced lipolysis in porcine adipocytes was applied as described by referred data (43, 44).

### Oil Red O staining

Cells were harvested on induction day 8. Cells were rinsed with Ca^2+^, Mg^2+^-free PBS twice and fixed in 4% polyoxymethylene in PBS (w/v) for 30 min at room temperature. Oil Red O (0.5 g; Amresco, Solon, OH) was dissolved in isopropanol (100 ml, w/v), diluted with water (6:4, v/v), and filtered. The fixed cells were then stained with the filtered Oil Red O solution for 1 h at room temperature, washed in water, and photographed.

### TG assay

Cells were washed with PBS and scraped from the plates in 200 μl lysis buffer per well. After being placed on ice for 5 min, the lysate was centrifuged at 8,000 *g*, 4°C for 1 min. The supernatant was analyzed by TG assay using the Triglyceride Assay Kit (APLLYGEN, Beijing, China) according to the manufacturer’s protocol with a series of diluted glycerol as a standard. Total protein detected by the BCA assay (Bioteke, Beijing, China) was used for normalization of TG concentration.

### miRNA microarray hybridization and data analysis

Total RNA was isolated from harvested cells by TRIzol reagent (Invitrogen, Carlsbad, CA) following the manufacturer’s instructions, 1 vol of isopropanol was used to precipitate RNA. Regarding both TNF-α treatment and control groups, total RNA samples from 6 wells of adipocytes were pooled together for miRNA expression profile analysis. RNA quality was assessed by using agarose gel electrophoresis and the NanoDrop 2000 spectrophotometer (Thermo Scientific, Waltham, MA). miRNA microarray analysis, including probe labeling, hybridization, hybridization image scanning, and initial data analysis, was performed by LC Sciences (Houston, TX). The miRNA microarray was based on Sanger miRBase release version 20. Normalization was performed using the cyclic locally weighted regression (LOWESS) method (45). Student’s *t*-test was used to assess statistically significant differences between treatment and control groups for microarray data.

### RNA extraction and real-time PCR

Total RNA was extracted from differentiated adipocytes using TRIzol reagent. RNA density was determined using the NanoDrop 2000. Total RNA (2 μg) was reverse-transcribed to cDNA using Moloney Murine Leukemia Virus reverse transcriptase (Promega, Madison, WI) with OligodT18 (IR) or Universal Adaptor Primer (miRNAs) from the One Step PrimeScript^®^ miRNA cDNA Synthesis Kit. After 1 h of incubation at 42°C and 10 min of deactivation at 75°C, the reaction mixes were used as the templates for PCR. Real-time PCR was performed with standard protocols on the STRATAGENE Mx3005P sequence detection system. The PCR mixture contained 2 μl of cDNA, 10 μl of 2× SYBR Green PCR Master Mix, 0.3 μl of each primer, and water to make up the final volume to 20 μl. The reaction was performed in a 96-well optical plate at 95°C for 1 min, followed by 35 cycles of 95°C for 15 s, optimal reannealing temperature for 15 s and 72°C for 40 s. All reactions were run in duplicate, and a negative control (NC) without template was included for each gene. Primers were designed based on the sequence of each gene by using Premier 5.0 (supplemental Table S1).

### Chromosomal localization

Based on mature sequences of differentially expressed miRNAs in the microarray, miRNA precursor sequences were obtained from miRBase release 21 (http://www.mirbase.org). These sequences were then mapped to the pig genome (sscorfa10.2; http://www.ensembl.org/Sus_scrofa/), and the chromosomal localization map was plotted according to their relative position on the chromosome.

### Target prediction and pathway analysis

We predicted target genes of miRNA in pigs at the genome level. In brief, the 3′-untranslated region (UTR) sequences of porcine transcripts in the whole genome were obtained from Ensembl genome browser 80 (sscorfa10.2; http://www.ensembl.org/Sus_scrofa/). Mature differentially expressed miRNAs sequences were downloaded from miRBase release 21 (http://www.mirbase.org), and RNAhybrid software (http://www.bibiserv.techfak.uni-bielefeld.de/rnahybrid) was used to analyze miRNA targets by using its own algorithm. Our prediction was restricted to a perfect match of the seed region (2–7 bases of the miRNA 5′ end; G:U matches were permitted), due to the importance of the seed sequence for miRNA-mRNA binding (46). In addition, the matches were restricted to those with less than 220 kcal/mol of low free energy in the binding of miRNA-mRNA. Furthermore, the Database for Annotation, Visualization and Integrated Discovery (DAVID) v6.7 online service (http://david.abcc.ncifcrf.gov/) (47) was used for Gene Ontology (GO) analysis and Kyoto Encyclopedia of Genes and Genomes (KEGG) pathway analysis based on all differentially expressed miRNA potential targets, and the minimum number of genes of potential pathways affected by differentially expressed miRNAs was 20. Individual GO analysis and KEGG analysis of miR-146a-5p were also conducted based on the potential targets of miR-146a-5p.

### Protein extraction and Western blot

Cells were lysed with RIPA buffer with protease inhibitors. Total soluble protein was quantified by the BCA protein assay. Total protein (30 μg) was loaded onto a 10% SDS-PAGE gel, separated by electrophoresis, and transferred onto a polyvinylidene difluoride membrane. Blots were blocked with 5% skim milk and incubated with primary antibody overnight at 4°C, followed by incubation with secondary antibody for 1 h at room temperature, and measured with an Infrared Imaging System (LI-COR, Lincoln, NE). Protein expression was normalized by detection of β-actin (Abcam, Cambridge, UK). Protein from cells treated with TNF-α/DMEM-F12 and inducted to day 8 was isolated to measure the expression level of IR.

### Transient miRNA transfection

Primary preadipocytes were cultured in 6-well plates. Cells at 80–90% confluency were used for transfections. Upregulation of miR-146a-5p was achieved by transfecting cells with 100 pmol per well of synthetic RNA duplex (mimics; GenePharma, Guangzhou, China) and NC (GenePharma) according to the manufacturer’s protocol. Sequences of the miR-146a-5p mimics were 5′-UGAGAACUGAAUUCCAUGGGUU-3′ (48) and 5′-CCCAUGGAAUUCAGUUCUCAUU-3′ (antisense), and those of the NC were 5′-UUCUCCGAACGUGUCACGUTT-3′ (48) and 5′-ACGUGAC­A­CGUUCGGGAATT-3′ (antisense). Similarly, inhibition of miR-146a-5p was achieved by transfecting cells with 100 pmol per well of 29-O methylated single-stranded miR-146a-5p antisense oligonucleotides (hereafter referred to as “inhibitor” GenePharma), The sequence of the miR-146a-5p inhibitor was 5′- AACCCAUGGAAUUCAGUUCUCA-3′, and that of the negative control for the inhibitor (iNC; GenePharma) was 5′-CAGUACUUUUGUGUAGUACAA-3′. At 6 h following transfection, media were replaced by induction medium every other day. On induction day 4, 6, and 8, cells from 1 well of each group (mimics group, NC group, inhibitor group, or iNC group) were used to conduct Oil Red O staining (see Oil Red O staining), and the remaining cells were used to conduct the TG assay (see TG assay) and Western blot of IR and phospho-IRS-1 (see Protein extraction and Western blot).

### Dual-luciferase reporter assay

The 3′-UTR sequences of porcine transcripts in the whole genome were obtained from the Ensembl genome browser 80 (sscorfa 9; http://www.ensembl.org/Sus_scrofa/). The 3′-UTR of IR contains the highly conserved binding sites of miR-146a-5p, and the sequence containing the binding sites (84 bp) is as follows: 5 ’-CTCGAGCTCA­CTCCCAAGTTCTCTTACTAGGCA­GGGTCCACAACTAGCCTCCAGTC

ACATTTTCCTTTGGGCATGAGCTCTAGA-3′. Furthermore, the 3′-UTR sequence was inserted into the pmirGLO Vector (Promega) with *Xho*I and *Xba*I double digestion to construct the recombinant dual-luciferase reporter vector, pmirGLO. Meanwhile, a plasmid containing the mutant IR 3′-UTR, pmirGLO-Mut, was generated by mutating the core sequence of the miR-146a-5p binding sites through DNA synthesis (Sangon Biotech Co. Ltd., Shanghai, China), and the sequence was as follows: 5′-CTCGAGCTCACTCCCACACCACATTACTAGGCAGGGTCCACAACTAGCCTCCAGTCACATTTTCCTTTGGGCATGAGCTCTAGA-3′. Similarly, a plasmid containing the deleted IR 3′-UTR, pmirGLO-Del, was generated by deleting the core sequence of miR-146a-5p binding sites through DNA synthesis, and the sequence was as follows: 5′-CTCGAGCTCACTCCCATTACTAGGCAGGGTCCACA­A­C­TAGCCTCCAGTCACATTTTCCTTTGGGCATGAGCTCTAGA-3′. Sequences of pmirGLO dual-luciferase reporter vectors are shown in supplemental Table S2. Chinese hamster ovary (CHO) cells were maintained in RPMI-1640 (GIBCO) and supplemented with 10% FBS (GIBCO). Lipofectamine 2000 (Invitrogen) was used for transfections. Cells (4 × 10^4^ per well) were plated in a 96-well plate. At 60–70% confluency, cells were transfected with 3 pmol miR-146a-5p mimics/NC and 100 ng pmirGLO/pmirGLO-Mut/pmirGLO-Del. Cells were collected 48 h after transfection, and luciferase activity was measured with the Dual-GLO luciferase reporter assay system (Promega).

### IR siRNA design and transfection

IR siRNA-1 (sense: 5′-CCGACUCACAGAUCCUCAATT-3′, antisense: 5′-UUGAGGAUCUGUGAGUCGGTT-3′), IR siRNA-2 (sense: 5′-GCGUCACUUCACUGGCUAUTT-3′, antisense: 5′-AUA­GCCAG­­UG­AAGUGACGCTT-3′), IR siRNA-3 (sense: 5′-GGAC­CAUUGU­A­UGCUUCUUTT-3′, antisense: 5′-AAGAA­GCA­UACAAUGGUCCTT-3′), IR siRNA-4 (sense: 5′-GGACCAUUGU­AUGCUUCUUTT, antisense: 5′-AAGAAGCAUACAAUGGU­C­­C­TT-3′), and the NC (sense: 5′-UUCUCCGAACGUGUCA­C­­GUTT-3′, antisense: 5′-ACGUGACACGUUCGGAGAATT-3′) were designed and synthesized by GenePharma. Preadipocytes were transfected with IR siRNAs or NC, and the most effective siRNA was chosen for subsequent analysis. After transfection with the most effective siRNA or NC, cells were induced to differentiate and collected at induction day 8 for Western blot analysis of IR (see “Protein extraction and Western blot”).

### Statistical analysis

All experiments included at least five replicates per group. Data were evaluated by Student’s *t*-test (real-time PCR, TG assay, glucose clearance assay) or one-way ANOVA analysis (siRNA experiment), and differences between groups were considered statistically significant at *P* < 0.05. All statistical analyses were performed with PASW Statistics 18 software.

## RESULTS

### TNF-α inhibits adipogenesis in porcine preadipocytes

**Fig. 1.**
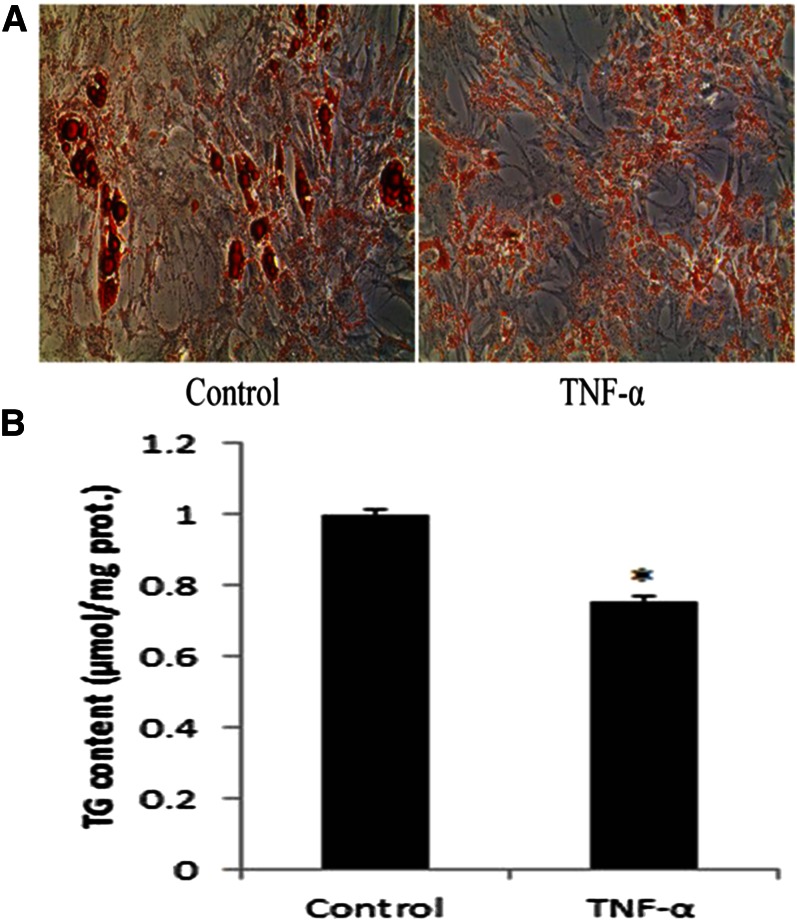
TNF-α inhibits adipogenesis of porcine preadipocytes. Preadipocytes were treated with TNF-α, and cells were collected on induction day 8 when reaching mature status. A: Formation of lipid droplets in cells treated with TNF-α was notably inhibited when compared with control group by staining with Oil Red O. Scale bars, 100 μm. B: Inhibition of adipogenesis was also determined by measuring the TG level and adjusted by protein content. Each sample was assayed in duplicate (* *P* < 0.05, n = 5).

**Fig. 2.**
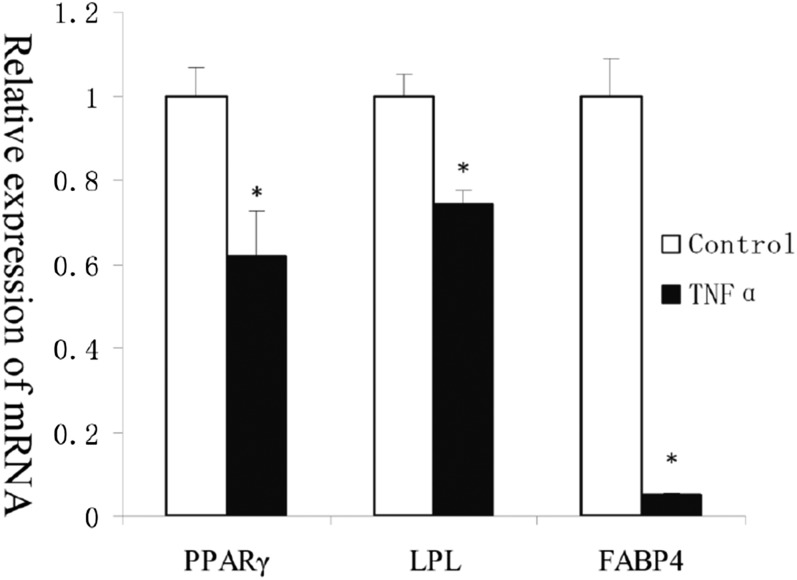
TNF-α inhibits expression of mature adipocyte markers during induced adipogenesis. Preadipocytes were treated with a 100 ng/ml dose of TNF-α, and cells were collected on induction day 8. mRNA expression levels of FABP4, PPARγ, and LPL were tested with quantitative RT-PCR method. These mature adipocyte markers were significantly decreased during induced adipogenesis with addition of TNF-α. Each sample was assayed in duplicate (* *P* < 0.05, n = 5).

In order to explore the effect of TNF-α on adipogenesis of porcine preadipocytes, cells were treated with TNF-α (100 ng/ml) and collected on induction day 8. Results of the Oil Red O assay (**Fig. 1A**) showed that TNF-α inhibited adipogenesis. To further verify this result, we conducted the TG assay (Fig. 1B), which similarly demonstrated that adipogenesis was significantly suppressed by TNF-α treatment. In addition, expression levels of mature adipocyte markers [such as fatty acid binding protein 4 (FABP4), PPARγ, and LPL] were also significantly decreased during induced adipogenesis with addition of TNF-α (**Fig. 2**). Together, these findings indicate that TNF-α inhibits adipogenesis of porcine preadipocytes.

### Differentially expressed miRNA profiles

**Fig. 3.**
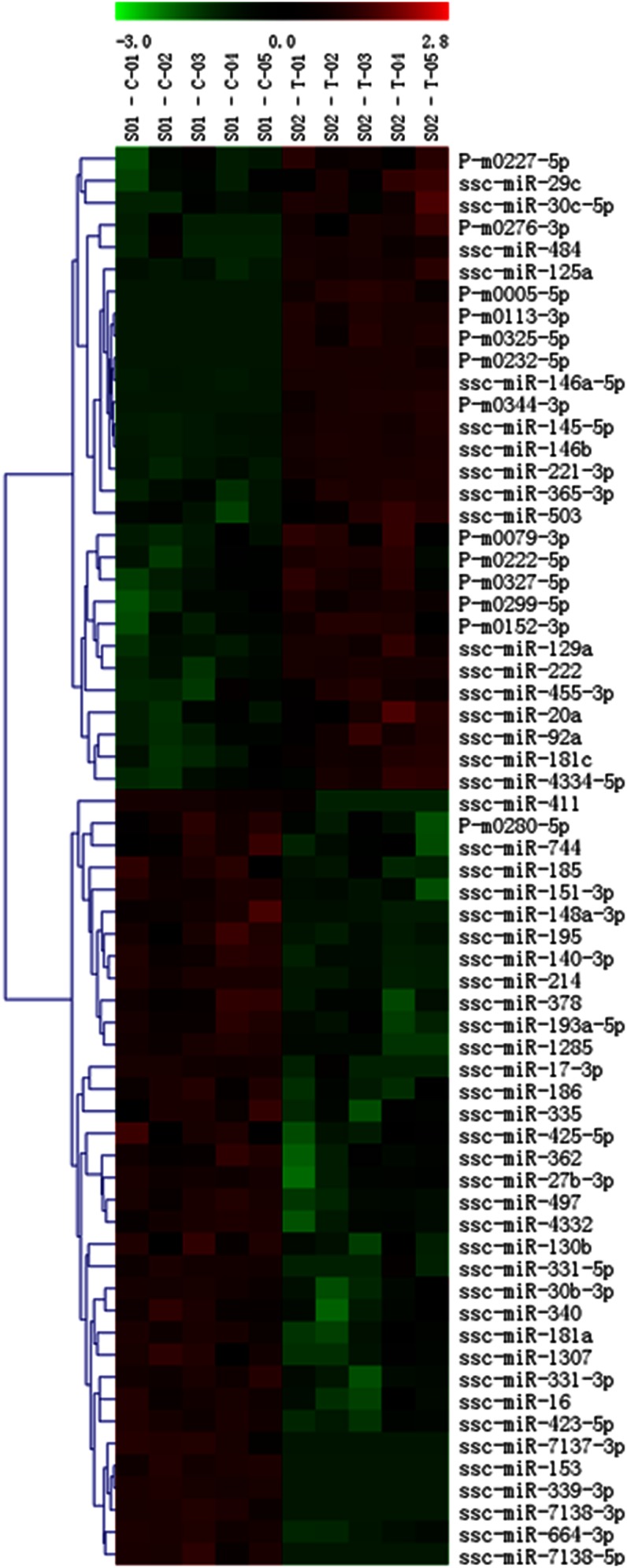
Heat map of miRNAs differentially expressed after TNF-α treatment. S01-C01-05 represents the control group, while S02-T01-05 represents the TNF-α group. Red denotes upregulated miRNAs, and green denotes downregulated miRNAs.

**Fig. 4.**
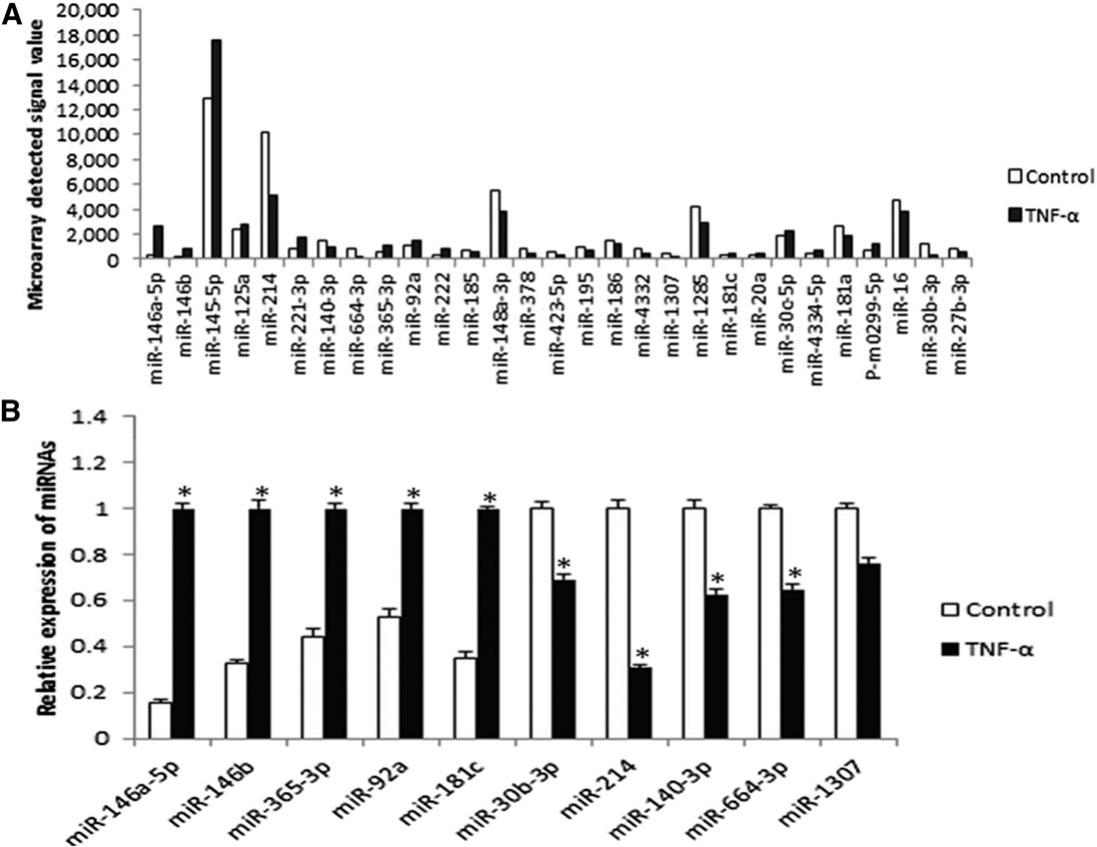
Differentially expressed miRNA profiles. A: Degree of up- and downregulation of the 29 significantly expressed miRNAs with strong signal values (signal ≥500). B: Real-time PCR analysis of randomly selected miRNAs from microarray assay. U6 was used as an internal reference (* *P* < 0.05, n = 6).

To examine miRNA expression profiles of porcine adipocytes treated with TNF-α, a high-throughput miRNA microarray was conducted (**Fig. 3**). After normalization and quality assessment, 637 of 719 probes were detected. Among them, 29 miRNAs were significantly differentially expressed with strong signal intensity (signal ≥500), while 35 were clearly expressed but with weak signal intensity (signal <500). Upregulated and downregulated amplitudes are shown in **Fig. 4A**. Among the 29 significantly differentially expressed miRNAs with strong signal values, 13 of them were remarkably upregulated, and 16 of them were intensely downregulated. miR-146a-5p and miR-146b showed the highest upregulation levels of 10.24-fold and 13.62-fold, respectively, in the TNF-α group. The fact that miR-146a-5p exhibited a higher signal value indicated that it generally was expressed at a higher average degree than miR-146b in normal porcine adipocytes. Thus, miR-146a-5p was chosen for subsequent analysis with the assumption that it plays an essential role during the process of TNF-α-stimulated inhibition of adipogenesis.

The 29 differentially expressed miRNAs were mapped to pig chromosomes (sscorfa10.2). Among them, five miRNAs (miR-365-3p, miR-378, miR-30c-5p, miR-181a, and miR-92a) had two copies in the genome, while P-m0299-5p did not map to any chromosome in the database available (supplemental Table S3).

### Verification of microarray results by real-time PCR

Five upregulated (miR-146a-5p, miR-146b, miR-365-3p, miR-92a, and miR-181c) and five downregulated (miR-30b-3p, miR-214, miR-140-3p, miR-664-3p, and miR-1307) miRNAs from the microarray were selected randomly to conduct real-time PCR. As shown in Fig. 4B, results of the real-time PCR were fully consistent with those of the microarray.

### Target prediction and pathway analysis

TargetScan, RNAhybrid ,and miRanda were used to predict targets of each of the 29 differentially expressed miRNAs, and an intersection of the 4,106 targets was obtained consequently (supplemental Table S4). KEGG pathway analysis and GO analysis were conducted by using the DAVID v6.7 online service. The GO analysis of predicted targets of the 29 differentially expressed miRNAs and the main predicted biological processes, cellular components, and molecular functions in which the 29 miRNAs are involved are shown in supplemental Fig. S1. The KEGG pathway analysis revealed that the predicted targets of the 29 differentially expressed miRNAs participate in 57 pathways, 3 (insulin signaling pathway, adipocytokine signaling pathway, and type 2 diabetes mellitus pathway) of which are relevant to adipogenesis. The predicted targets included genes encoding crucial adipogenesis-related molecules such as IR, protein kinase B (Akt), phosphatidylinositol 3-kinase, TNF, MAPK, and mammalian target of rapamycin(mTOR).

**Fig. 5.**
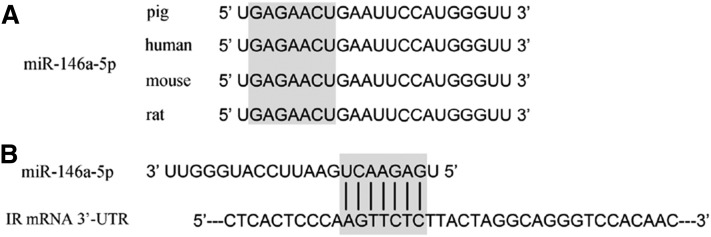
Conserved analysis of miR-146a-5p and its target gene across species. A: Mature sequences of miR-146a-5p in the pig, human, mouse, and rat genomes. Gray area denotes seed sequences. B: Binding site of IR mRNA 3′-UTR and miR-146a-5p seed region.

We further conducted KEGG pathway analysis on predicted targets of miR-146a-5p individually because it was expressed quite highly after TNF-α treatment. miR-146a-5p was predicted to participate in 21 pathways (supplemental Table S5). Target prediction identified IR, an essential factor in the insulin signaling pathway, as one of the potential targets of miR-146a-5p. Therefore, we speculated that miR-146a-5p targeting of IR would impact the TNF-α-stimulated inhibition of adipogenesis. Interestingly, miR-146a-5p was found to be highly conserved among pigs, humans, mice, and rats (**Fig. 5A**). The 3′-UTR of IR mRNA contains a binding site that perfectly matched the seed region of miR-146a-5p (Fig. 5B).

### TNF-α suppresses IR mRNA and protein expression

**Fig. 6.**
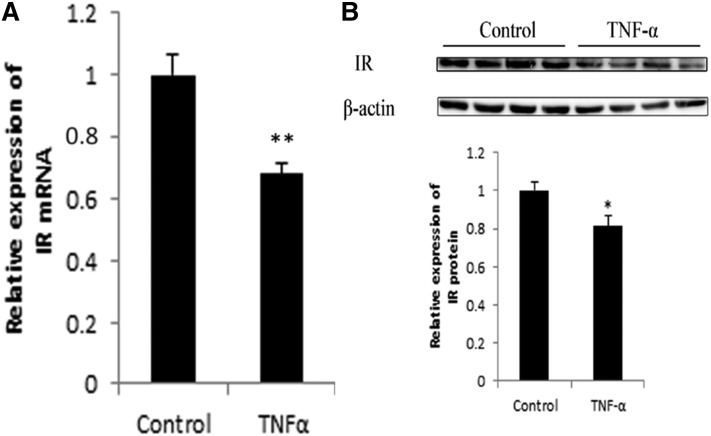
TNF-α suppresses IR mRNA and protein expression. A: Real-time PCR analysis of IR mRNA after TNF-α treatment. β-actin was used as an internal reference (** *P* < 0.01, n = 6). B: Western blot and gray-scale scanning analysis of IR after TNF-α treatment (* *P* < 0.05, n = 4).

The KEGG pathway analysis revealed that nearly all of the differentially expressed miRNAs (28 out of 29 miRNAs) were predicted to participate in the insulin signaling pathway. As an important factor in the insulin signaling pathway, we focused our attention on the IR expression level after TNF-α treatment. We treated preadipocytes with TNF-α and stimulated the cells to mature status. Real-time PCR analysis (**Fig. 6A**) revealed that TNF-α significantly inhibited IR mRNA expression, and the Western blot results (Fig. 6B) also showed that TNF-α significantly reduced the IR protein level. 

### miR-146a-5p inhibits adipogenesis

**Fig. 7.**
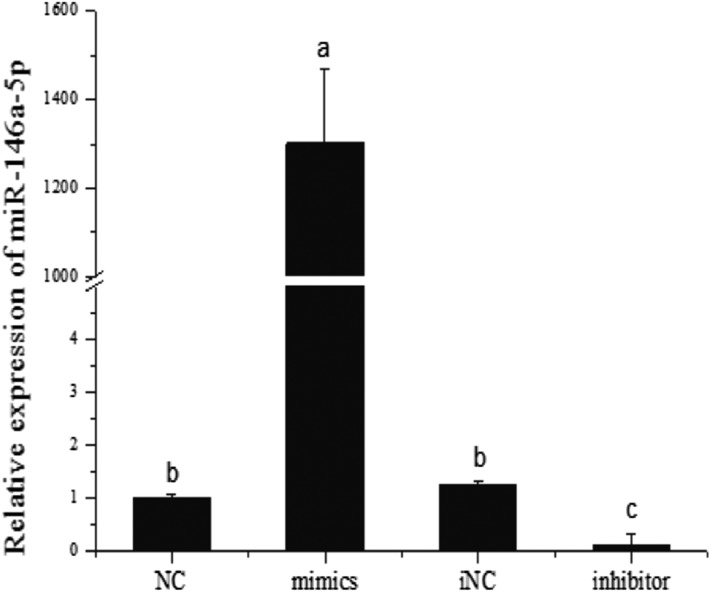
The endogenous miR-146a-5p levels with transfection of miRNA mimics or inhibitor. miR-146a-5p mimics/NC/inhibitor/iNC were transfected into preadipocytes, and cells were collected on day 8 postinduction. After transfection and day 8 postinduction with miR-146a-5p mimics and inhibitor, the expression levels of miR-146a-5p were measured by a qPCR-based method. miRNA mimics and inhibitor significantly increased and decreased miR-146a-5p levels compare with NC and iNC control, respectively, in adipocytes. The means in each column followed by the different letters represent significant difference (*P* < 0.05), and the same letter represents nonsignificant difference (*P* > 0.05, n = 8).

**Fig. 8.**
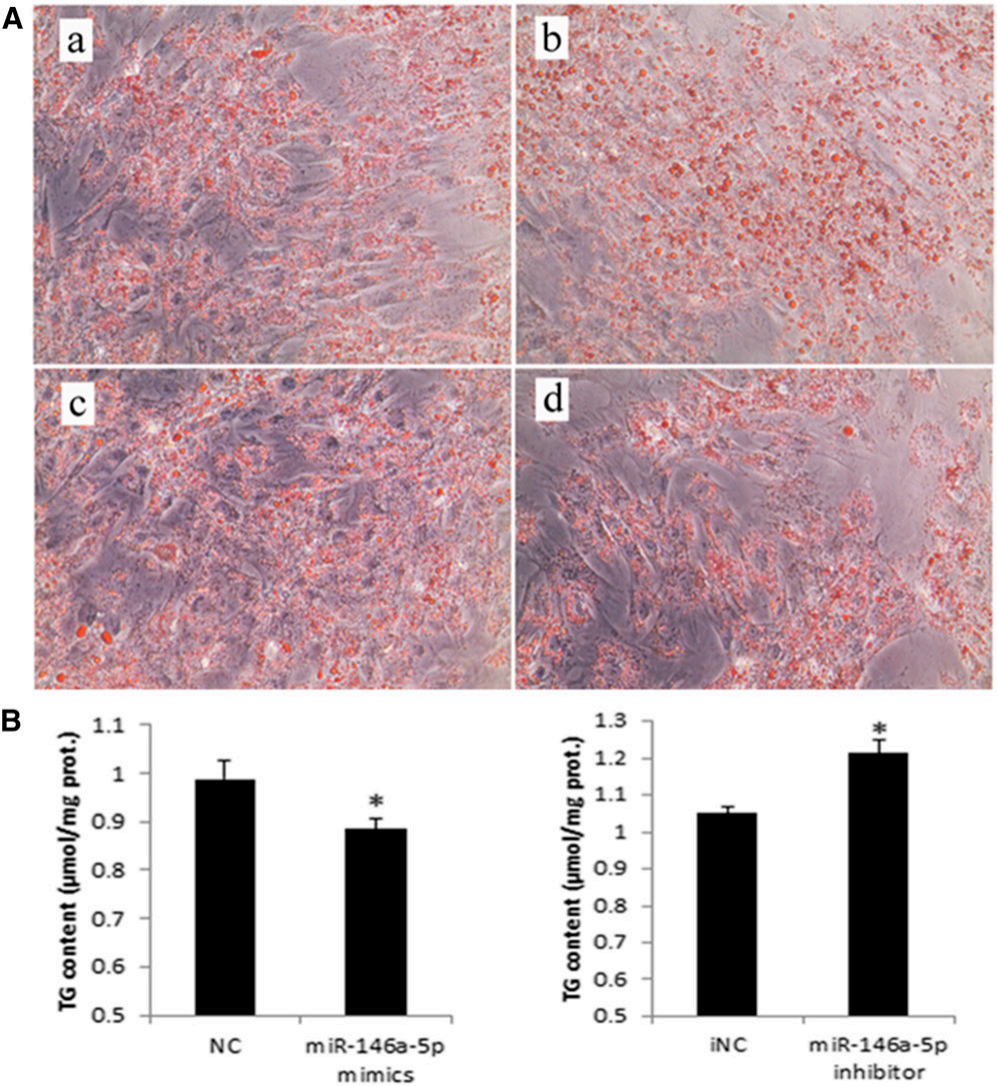
miR-146a-5p inhibits adipogenesis. Preadipocytes were transfected with miR-146a-5p mimics/NC/inhibitor/iNC. Cells were collected on induction day 8 when reaching mature status. A: Formation of lipid droplets in cells transfected with miR-146a-5p mimics (a), NC (b), inhibitor (c), and iNC (d) was observed by staining with Oil Red O. Scale bars, 100 μm. B: The corresponding TG level was also determined and adjusted by protein content. Each sample was assayed in duplicate (* *P* < 0.05, n = 5).

Because miR-146a-5p was increased dramatically after TNF-α treatment, we presumed that this miRNA could also inhibit adipogenesis of porcine preadipocytes. miR-146a-5p mimics/NC/inhibitor/iNC were transfected into preadipocytes, and cells were collected on day 8 postinduction. After transfection and day 8 postinduction with miR-146a-5p mimics and inhibitor, the expression levels of miR-146a-5p were measured by a quantitative PCR (qPCR)-based method. The results showed miRNA mimics and inhibitor significantly increased and decreased miR-146a-5p levels compare with NC and iNC control, respectively, in adipocytes (**Fig. 7**). miR-146a-5p mimics also obviously decreased lipid droplets in porcine adipocytes (**Fig. 8A-a**) when compared with the NC group (Fig. 8A-b), and this regulation was rescued by the miR-146a-5p inhibitor (Fig. 8A-c). In addition, the degree of differentiation was determined by the TG assay. Similar to the results of Oil Red O staining, miR-146a-5p mimics significantly reduced the amount of TG (Fig. 8B), and this effect was also rescued by transfection of the inhibitor (Fig. 8B).

### Verification of miR-146a-5p targeting 3′-UTR of IR mRNA by luciferase reporter assay

**Fig. 9.**
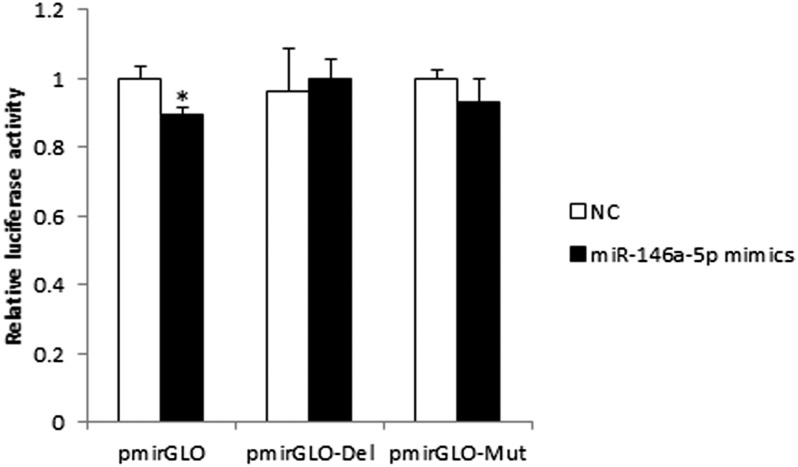
pmirGLO dual-luciferase reporter vectors analysis. CHO cells were transfected with each of the constructed plasmids, together with miR-146a-5p mimics/NC (* *P* < 0.05, n = 8). Relative luciferase activity was calculated by firefly luminescence/Renilla luminescence.

To verify IR, a crucial factor in the insulin signaling pathway, as a target of miR-146a-5p, we constructed three pmirGLO dual-luciferase reporter vectors: pmirGLO (wild-type 3′-UTR of IR mRNA with seed sequence), pmirGLO-Mut (3′-UTR of IR mRNA with seed sequence muted), and pmirGLO-Del (3′-UTR of IR mRNA with seed sequence deleted). CHO cells were then cotransfected with miR-146a-5p mimics/NC and pmiGLO/pmirGLO-Mut/pmirGLO-Del. Forty-eight hours after transfection, the luciferase activity of the wild-type group was assayed, and the miR-146a-5p mimics group showed lower luciferase activity when compared with the NC group. The reduction was rescued in the mutation group and deletion group (**Fig. 9**). Thus, IR was confirmed preliminarily as the target of miR-146a-5p.

### miR-146a-5p suppresses IR protein expression

**Fig. 10.**
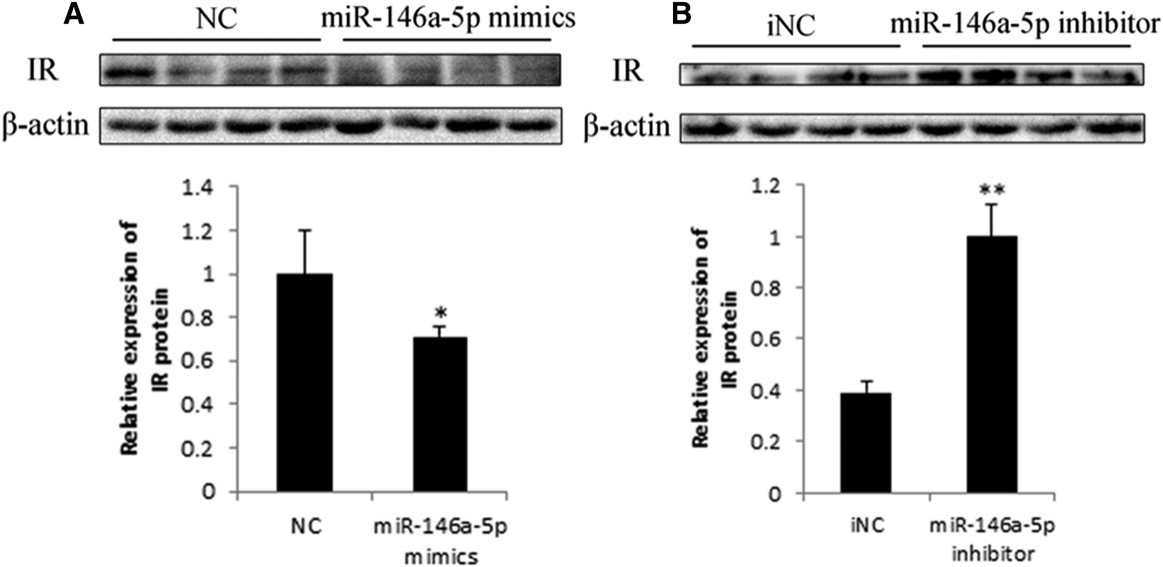
miR-146a-5p suppresses IR protein expression. Western blot and gray-scale scanning analyses of IR after transfection of miR-146a-5p mimics/NC/inhibitor/iNC (* *P* < 0.05, ** *P* < 0.01, n = 4).

To explore whether miR-146a-5p could affect the IR protein expression level, we transfected preadipocytes with miR-146a-5p mimics, NC, inhibitor, or iNC and measured IR protein expression on day 8 postinduction. Western blot analysis showed that miR-146a-5p mimics suppressed IR protein expression significantly (**Fig. 10A**), while the inhibitor rescued IR expression (Fig. 10B). These results further verified that miR-146a-5p targets the 3′UTR of IR mRNA, resulting in suppressed expression of IR protein expression.

**Fig. 11.**
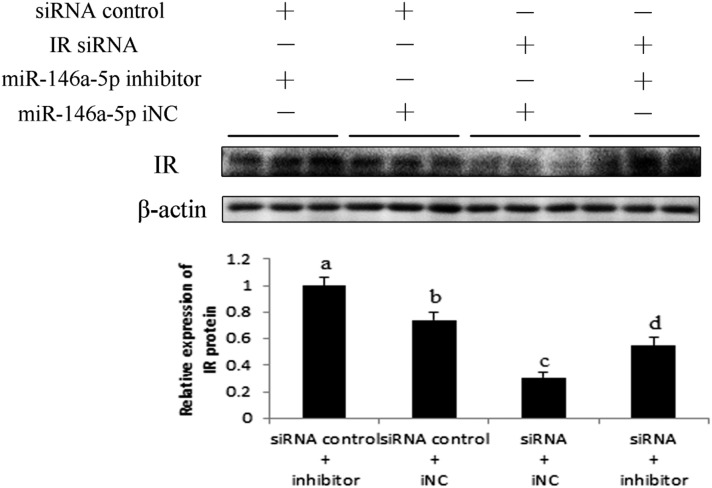
Western blot analysis of IR siRNA transfection. Preadipocytes were transfected with miR-146a-5p inhibitor/iNC and IR siRNA/siRNA control, and IR protein was assayed on induction day 8. Panels with different letters were considered statistically significant (*P* < 0.05, n = 3).

To further confirm the direct regulation of miR-146a-5p on IR, we transfected IR siRNA/siRNA control and miR-146a-5p inhibitor/iNC into preadipocytes and induced them to mature status. Cells were collected on day 8 postinduction, and then the IR protein level was determined by Western blot (**Fig. 11**). The IR siRNA + iNC group significantly reduced IR expression when compared with the siRNA control + iNC group, demonstrating specific inhibition of IR siRNA on the IR protein level. Furthermore, this inhibitory effect was rescued by addition of the miR-146a-5p inhibitor (IR siRNA + inhibitor group). Together, these results further verified the miR-146a-5p targeting of IR.

### miR-146a-5p downregulates IRS-1 tyrosine phosphorylated protein expression

**Fig. 12.**
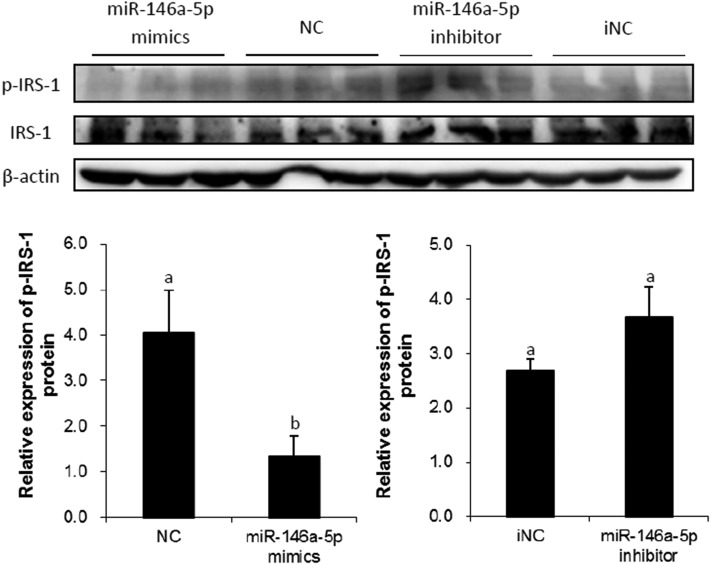
Western blot analysis of tyrosine phosphorylation of IRS-1. Preadipocytes were transfected with miR-146a-5p mimics/NC/inhibitor/iNC, and tyrosine phosphorylation of IRS-1 was assayed on induction day 8 after treating with 100 nM insulin for 1 h at 37°C. IRS-1 was used to adjust the expression of tyrosine phosphorylation of IRS-1. The means in each column followed by the different letters represent significant difference (*P* < 0.05), and the same letter represents nonsignificant difference (*P* > 0.05, n = 3).

IRS-1 is an important factor in the insulin signaling pathway, and TNF-α diminishes insulin-induced tyrosine phosphorylation of IRS-1 during the process of insulin resistance (49). Thus, we transfected miR-146a-5p mimics/NC/inhibitor/iNC into preadipocytes and induced the cells to mature status (see Materials and Methods) when the expression of IRS-1 tyrosine phosphorylated protein was assayed by Western blot. The results showed that miR-146a-5p mimics inhibited the tyrosine phosphorylation of IRS-1, while the miR-146a-5p inhibitor rescued this trend (**Fig. 12**). 

## DISCUSSION

Adipose tissue is well accepted as the major energy reserve in the body. Overloading of white adipose tissue beyond its storage capacity may lead to lipid disorders such as obesity. Obesity is closely associated with pathological disorders, including diabetes, hypertension, and cancer. TNF-α has been a research focus during the past few decades as a potent cytokine in adipose biology and is involved in a myriad of biological processes. TNF-α is a key mediator of inflammatory reactions, and it can cause tumor cell necrosis (30). It has also been reported to regulate adipocyte metabolism at numerous sites, including hormone receptor signaling, fatty acid metabolism, glucose metabolism, and transcriptional regulation (32). Targeting TNF-α has been suggested to be a potential therapeutic method for insulin resistance and type 2 diabetes (31). miR-145 regulates adipocyte lipolysis by increasing production and processing of TNF-α in human fat cells (49). In our previous study, we discovered that miR-181a targets TNF-α, and suppression of miR-181a decreased the expression of fat synthesis-associated genes phosphodiesterase 3B, LPL (50), PPARγ, glucose transporter 1 (GLUT1), GLUT4, adiponectin, and FASN, as well as key lipolytic genes hormone-sensitive lipase and ATGL (51). Liu and et al. (52) found that TNF-α treatment led to the upregulation of miR-155 through the nuclear factor κB (NFκB) pathway in 3T3-L1 cells, and miR-155 targets 3′-UTRs of C/EBPβ and cAMP response element binding protein(CREB). However, the mechanism of miRNAs in the process of TNF-α-stimulated inhibition of adipogenesis remains deficient.

In this study, we detected 29 differentially expressed miRNAs after TNF-α treatment by miRNA microarray. Among the 29 differentially expressed miRNAs, 13 were upregulated and 16 were downregulated. KEGG analysis of the 29 differentially expressed miRNAs revealed that the predicted adipogenesis relevant pathways in which they participate include fatty acid metabolism pathway, ether lipid metabolism pathway, mTOR signaling pathway, PPAR signaling pathway, Toll-like receptor (TLR) signaling pathway, MAPK signaling pathway, type 2 diabetes mellitus pathway, p53 signaling pathway, insulin signaling pathway, and adipocytokine signaling pathway. Target prediction of the 29 differentially expressed miRNAs revealed that miR-148a-3p, miR-27b-3p, miR-423-5p, miR-125a, miR-181c, and miR-365-3p target TNF-α. The miR-148 family, including miR-148a and miR-148b, has been reported to be negative regulators of the innate immune response, which in turn inhibit the production of cytokines including TNF-α in dendritic cells (53). The downregulation of miR-148a-3p after TNF-α treatment in our microarray analysis was an interesting finding. Potentially, miR-148a-3p also participates in immune activity in porcine adipocytes and forms a loop with TNF-α. Jin et al. (54) found that TNF-α promotes miR-27b-3p expression through the AKT/NFκB signaling cascade in human breast cancer cell lines. However, in our study, miR-27b-3p was detected to be downregulated after TNF-α treatment in porcine adipocytes. Thus, miR-27b-3p may predominantly function as a promoter of tumor growth in cancer cells, while it mainly participates in adipogenic processes in adipocytes.

Expression levels of miR-146a-5p and miR-146b were the most drastically changed at 10.24-fold and 13.62-fold, respectively, by TNF-α treatment of adipocytes. Because the signal value of miR-146a-5p was higher than that of miR-146, we assumed that miR-146a-5p would be expressed at a higher level than that of miR-146b in adipocytes. Therefore, miR-146a-5p was chosen for further analysis in this study. miR-146a-5p was predicted to target IR, which is a crucial factor in the insulin signaling pathway. We initially found that miR-146a-5p suppressed adipogenesis via the Oil Red O assay and TG assay. Use of the pmirGLO dual-luciferase reporter vectors initially indicated that miR-146a-5p targets the 3′-UTR of the IR mRNA. Western blot results also showed that miR-146a-5p could inhibit the expression of IR protein, suggesting a regulatory role of miR-146a-5p on its target. The subsequent IR siRNA assay further confirmed that miR-146a-5p specifically targets IR. It is possible that miR-146a-5p inhibits adipogenesis through targeting IR. Our study provided the first evidence of miR-146a-5p targeting IR. Kanety et al. (55) found that TNF-α diminishes insulin-induced tyrosine phosphorylation of IRS-1 during the process of insulin resistance. We transfected adipocytes with miR-146a-5p mimics, and tyrosine phosphorylation of IRS-1 was determined to be downregulated by Western blot. These results suggest that miR-146-5p participates in TNF-α-induced insulin resistance and the insulin signaling pathway.

miR-146a has been reported to be involved in papillary thyroid carcinoma (56), rheumatoid arthritis (57), pancreatic cancer (58), inflammatory reaction (59), and innate immune responses (60). Cho et al. (61) discovered that overexpression of miR-146a could inhibit basal and TNF-α- and TLR ligand-induced osteogenic differentiation. In MSCs, diazoxide was shown to potentiate cell survival via NFκB-dependent miR-146a expression by targeting Fas (62). In vascular smooth muscle cells, miR-146a targets Kruppel-like factor 4(KLF4) 3′-UTR and has an important role in promoting cell proliferation (63). Perng et al. (64) found that miRNA-146a expression could positively regulate TNF-α-induced interleukin-8 production in MSCs and differentiated lung epithelial-like cells. In marrow-derived MSCs, miR-146a was shown to play a critical role in the control of the immunoregulatory potential of bone tissue by targeting prostaglandin E_2_ synthase (ptges-2) (36). However, no evidence of the function of miR-146a-5p in adipogenesis in primary adipocytes has been presented until now. Our finding is the first to explain the role that miR-146a-5p potentially plays in the process of adipogenesis. Based on the finding that miR-146a-5p inhibits adipogenesis, it can be a potential target of obesity treatment in the future.

**Fig. 13.**
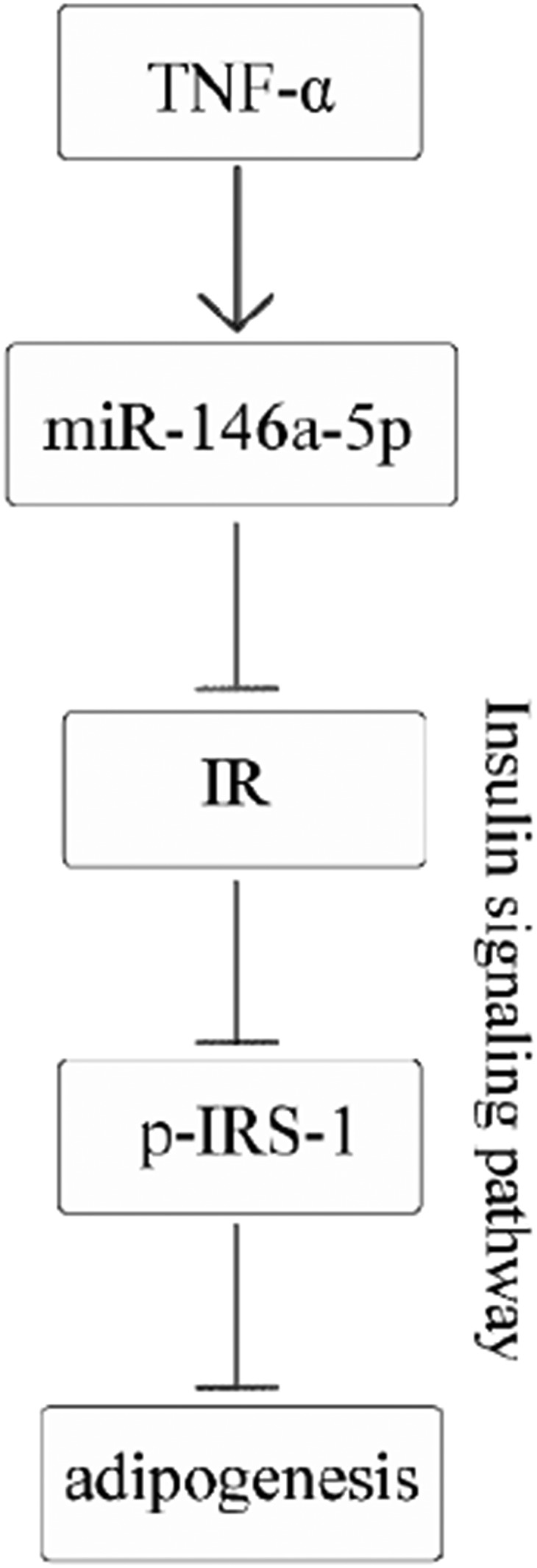
Summary of regulation of TNF-α-miRNA-insulin signaling pathway. TNF-α improves expression of miR-146a-5p (↓), and miR-1465p inhibits adipogenesis by targeting IR (⊥).

TNF-α was confirmed to inhibit adipogenesis and shown to suppress IR at both the mRNA and protein levels. TNF-α treatment caused changes in the expression profiles of 29 miRNAs. These differentially expressed miRNAs were considered mediators of TNF-α-stimulated inhibition of adipogenesis and downregulation of IR. We focused our study on miR-146a-5p and found that it inhibited adipogenesis through targeting IR. miR-146a-5p also reduced the tyrosine phosphorylation of IRS-1, indicating that this miRNA participates in the insulin signaling pathway (**Fig. 13**). 

Pigs serve as an excellent biomedical model for human studies because the pig genome has high sequence and chromosome structural homology with humans (3). Pigs have been used to construct biomedical models (65, 66), genomics and melanoma models (67, 68), infectious disease models (69–71), and antiviral response models (72–74) due to their many advantages in research (3). As far as we know, hsa-miR-146a-5p (human miR-146a-5p) exactly shares the sequence 5′-UGAGAACUGAAUUCCAU­G­G­G­UU-3′ with ssc-miR-146a-5p and mmu-miR-146a-5p. Moreover, 3′-UTR of human IR mRNA contains the same sequence of our verified ssc-miR-146a-5p binding site (AGTTCTC) (Fig. 5). According to many preferentially conserved sites that match the miRNA seed (nucleotides 2–7), particularly those in 3′-UTRs, most mammalian mRNAs are conserved targets of miRNAs (75). It has been reported that miR-144 directly inhibits IRS-1, a key molecule in insulin signaling in rat and human based on the prediction that miR-144 binds to the 3′UTR of IRS-1, and this targeting relationship confirmed in 3T3-L1 adipocytes and HeLa cells, respectively (76). In addition, the regulatory function of human miRNAs was often used for investigations on the mouse and rat model because of same miRNA mature sequence (77). In our lab, ssc-miR-130a from pigs was approved to target the PPARγ gene as described in human and mouse (78). Thus, it is believed that miR-146a-5p regulation in pigs will be an important animal model for the study of diabetes in humans and a theoretical basis for research in adipogenesis in humans in the future.

## Supplementary Material

Supplemental Data
