# Supplementary material for: miR-146a-5p inhibits TNF-α-induced adipogenesis via targeting insulin receptor in primary porcine adipocytes
Source: J Lipid Res. 2016 Aug;57(8):1360–72. doi: 10.1194/jlr.M062497 (PMC4959853; doi:10.1194/jlr.M062497)
Supplement: Supplemental Data [file supp_57_8_1360__index.html]

MiR-146a-5p inhibits TNF-α-induced adipogenesis via targeting insulin receptor in primary porcine adipocytes — miR-146a-5p inhibits TNF-α-induced adipogenesis via targeting insulin receptor in primary porcine adipocytes — Supplemental Data 

# miR-146a-5p inhibits TNF-α-induced adipogenesis via targeting insulin receptor in primary porcine adipocytes

## Supplemental Data

- supl. figures and tables (.doc, 300 KB) - supl. figures and tables
- supl table s4 (.xls, 210 KB) - supl table s4
