# Supplementary material for: miR-146a-5p inhibits TNF-α-induced adipogenesis via targeting insulin receptor in primary porcine adipocytes
Source: J Lipid Res. 2016 Aug;57(8):1360–72. doi: 10.1194/jlr.M062497 (PMC4959853; doi:10.1194/jlr.M062497)
Supplement: Supplemental Data [file 10.1194_M062497_jlr.M062497-1.doc]

Supplementary Figures and Tables:

Supplementary Figure S1: GO analysis results of predicted targets of the 29 differentially expressed miRNAs.

Supplementary Table S1: Primers of INSR and microRNAs.

Supplementary Table S2: The sequences of pmirGLO Dual-Luciferase reporter vectors.

Supplementary Table S3: Chromosome location of miRNAs.

Supplementary Table S5: KEGG pathway analysis of miR-146a-5p.

Supplementary Figure S1: GO analysis results of predicted targets of the 29 differentially expressed miRNAs.


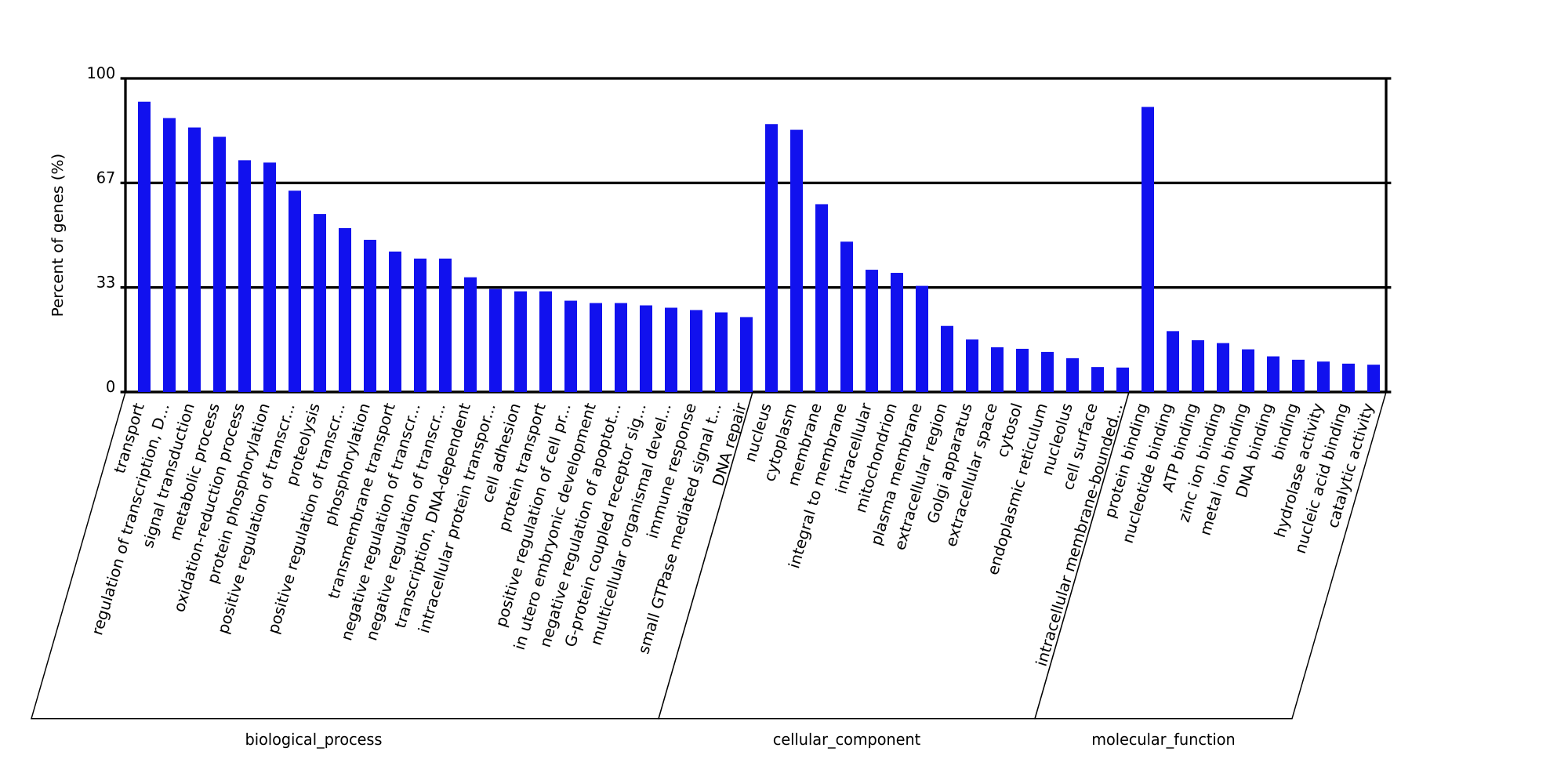


Supplementary Table S1: Primers of INSR and microRNAs.

Table S1-1 Primers of INSR and β-actin

| Gene | Primer sequence (5’-3’) | | Products（bp） | Tm（℃） |
| --- | --- | --- | --- | --- |
| INSR | Forwards | GCCCTGTGACCCATGAAATC | 215 | 53 |
|  | Reverse | CCGAACTCGAACGCTGTAAT |  |  |
| β-actin | Forwards | CCACGAAACTACCTTCAACTC | 131 | 56 |
|  | Reverse | TGATCTCCTTCTGCATCCTGT |  |  |

Table S1-2 Primers of microRNAs

| MicroRNA | Primer sequence(5’-3’) | Products（bp） | Tm（℃） |
| --- | --- | --- | --- |
| ssc-miR-146a-5p | GTGAGAACTGAATTCCATGGGTT | 79 | 60 |
| ssc-miR-146b | CAGTGAGAACTGAATTCCATAGGC | 80 | 60 |
| ssc-miR-365-3p | CGTAATGCCCCTAAAAATCCTT | 78 | 60 |
| ssc-miR-92a | TTACACTTGTCCCGGCCTGT | 76 | 60 |
| ssc-miR-181c | GAACATTCAACCTGTCGGTGAGT | 79 | 61 |
| ssc-miR-30b-3p | AGCTGGGAGGTGGATGTTTACT | 78 | 60 |
| ssc-miR-214 | ATACAGCAGGCACAGACAGGC | 77 | 60 |
| ssc-miR-140-3p | TACCACAGGGTAGAACCACGG | 77 | 60 |
| ssc-miR-664-3p | GCTATTCATTTATCTCCCAGCCTAC | 81 | 60 |
| ssc-miR-1307 | TATAACTCGGCGTGGCGTC | 75 | 60 |
| U6 | Forwards: CTCACTTCGGCAGCACATA  Reverse:AACTCTTCACGATTTTGTCTGTC |  | 58 |

Supplementary Table S2: The sequences of pmirGLO Dual-Luciferase reporter vectors.

| Name of vector | Sequence(5’-3’) |
| --- | --- |
| Wild type INSR 3’-UTR（sense） | TCGAGCTCGAGCTCACTCCCA***AGTTCTC***TTACTAGGCAGGGTCCACAACTAGCCTCCAGTCACATTTTCCTTTGGGCATGAGCTCTAGAT |
| Wild type INSR 3’-UTR（antisense） | CTAGATCTAGAGCTCATGCCCAAAGGAAAATGTGACTGGAGGCTAGTTGTGGACCCTGCCTAGTAA***GAGAACT***TGGGAGTGAGCTCGAGC |
| Mutated INSR 3’-UTR（sense） | TCGAGCTCGAGCTCACTCCCA***CACCACA***TTACTAGGCAGGGTCCACAACTAGCCTCCAGTCACATTTTCCTTTGGGCATGAGCTCTAGAT |
| Mutated INSR 3’-UTR（antisense） | CTAGATCTAGAGCTCATGCCCAAAGGAAAATGTGACTGGAGGCTAGTTGTGGACCCTGCCTAGTAA***TGTGGTG***TGGGAGTGAGCTCGAGC |
| Deleted INSR 3’-UTR（sense） | TCGAGCTCGAGCTCACTCCCATTACTAGGCAGGGTCCACAACTAGCCTCCAGTCACATTTTCCTTTGGGCATGAGCTCTAGAT |
| Deleted INSR 3’-UTR（antisense） | CTAGATCTAGAGCTCATGCCCAAAGGAAAATGTGACTGGAGGCTAGTTGTGGACCCTGCCTAGTAATGGGAGTGAGCTCGAGC |

Supplementary Table S3: Chromosome location of miRNAs.

| MiRNA Name | Chromosome location [+] | Chromosome location [-] |
| --- | --- | --- |
| ssc-miR-146a-5p | 16: 68488129-68488206 | 16: 68394520-68394597 |
| ssc-miR-146b | 14: 123301752-123301850 |  |
| ssc-miR-145-5p | 2: 157346127-157346212 |  |
| ssc-miR-125a | 6: 51858852-51858931 |  |
| ssc-miR-214 |  | 9: 126099461-126099570 |
| ssc-miR-221-3p |  | X: 45274873-45274942 |
| ssc-miR-140-3p | Scaffold GL892841.1:107277-107370 |  |
| ssc-miR-664-3p | 10: 11890513-11890592 |  |
| ssc-miR-365-3p |  | ssc-miR-365-1  3: 29976236-29976315 ssc-miR-265-2  12: 45152792-45152871 |
| ssc-miR-92a | ssc-miR-92a-1  11:66610762-66610841 | ssc-miR-92a-2  X: 126199512-126199591 |
| ssc-miR-222 |  | X: 45275613-45275692 |
| ssc-miR-185 | 14: 55086524-55086599 |  |
| ssc-miR-148a-3p | 18: 51216035-51216102 |  |
| ssc-miR-378 | ssc-miR-378-1  2: 157640353-157640432  ssc-miR-378-2  12: 38396700-38396767 |  |
| ssc-miR-423-5p |  | 12: 48287019-48287098 |
| ssc-miR-195 | 12: 54717315-54717394 |  |
| ssc-miR-186 | 6: 131558966-131559047 |  |
| ssc-miR-4332 |  | 6: 870,446-870,551 |
| ssc-miR-1307 |  | Scaffold GL895853.2: 46361-46440 |
| ssc-miR-1285 | 1: 200102137-200102216 | 1: 199694416-199694495 |
| ssc-miR-181c |  | 2: 65553664-65553750 |
| ssc-miR-20a | 11: 66610513-66610583 |  |
| ssc-miR-30c-5p |  | ssc-miR-30c-1  6: 157473595-157473674 ssc-miR-30c-2  1: 57721688-57721767 |
| ssc-miR-4334-5p | 15: 134433005-134433073 |  |
| ssc-miR-181a | ssc-miR-181a-2  1: 299322152-299322231 | ssc-miR-181a-1  10: 26427018-26427121 |
| ssc-miR-16 | ssc-miR-16-1  13: 108388290-108388366 |  |
| ssc-miR-30b-3p | 4: 6717643-6717718 |  |
| ssc-miR-27b-3p |  | 10: 31340880-31340959 |

Supplementary Table S5: KEGG pathway analysis of miR-146a-5p.

| Pathway | P value | Predicted target genes involved |
| --- | --- | --- |
| Cell cycle | 0.018 | 10 |
| Insulin signaling pathway | 0.094 | 10 |
| Small cell lung cancer | 0.005 | 9 |
| Apoptosis | 0.0062 | 9 |
| Dilated cardiomyopathy | 0.0086 | 9 |
| Neurotrophin signaling pathway | 0.043 | 9 |
| GnRH signaling pathway | 0.043 | 9 |
| Chronic myeloid leukemia | 0.0094 | 8 |
| Hypertrophic cardiomyopathy (HCM) | 0.018 | 8 |
| Hematopoietic cell lineage | 0.019 | 8 |
| T cell receptor signaling pathway | 0.056 | 8 |
| Lysosome | 0.079 | 8 |
| Fc epsilon RI signaling pathway | 0.037 | 8 |
| Pancreatic cancer | 0.026 | 7 |
| Arrhythmogenic right ventricular cardiomyopathy (ARVC) | 0.033 | 7 |
| Epithelial cell signaling in Helicobacter pylori infection | 0.055 | 7 |
| B cell receptor signaling pathway | 0.081 | 7 |
| VEGF signaling pathway | 0.081 | 7 |
| Adipocytokine signaling pathway | 0.061 | 6 |
| Non-small cell lung cancer | 0.065 | 6 |
| Type II diabetes mellitus | 0.061 | 5 |
